# scnanoseq: an nf-core pipeline for Oxford Nanopore single-cell RNA-sequencing

**DOI:** 10.1093/bioinformatics/btaf487

**Published:** 2025-09-04

**Authors:** Austyn Trull, Elizabeth A Worthey, Lara Ianov

**Affiliations:** Institutional Research Core Program—Biological Data Science Core, University of Alabama at Birmingham, Birmingham, AL, 35233, United States; Institutional Research Core Program—Biological Data Science Core, University of Alabama at Birmingham, Birmingham, AL, 35233, United States; Department of Genetics, University of Alabama at Birmingham, Birmingham, AL, 35233, United States; Institutional Research Core Program—Biological Data Science Core, University of Alabama at Birmingham, Birmingham, AL, 35233, United States; Department of Neurobiology, University of Alabama at Birmingham, Birmingham, AL, 35233, United States

## Abstract

**Motivation:**

Recent advancements in long-read single-cell RNA sequencing (scRNA-seq) have facilitated the quantification of full-length transcripts and isoforms at the single-cell level. Historically, long-read data would need to be complemented with short-read single-cell data in order to overcome the higher sequencing errors to correctly identify cellular barcodes and unique molecular identifiers. Improvements in Oxford Nanopore sequencing, and development of novel computational methods have removed this requirement. Though these methods now exist, the limited availability of modular and portable workflows remains a challenge.

**Results:**

Here, we present, nf-core/scnanoseq, a secondary analysis pipeline for long-read single-cell and single-nuclei RNA that delivers gene and transcript-level quantification. The scnanoseq pipeline is implemented using Nextflow and is built upon the nf-core framework, enabling portability across computational environments, scalability and reproducibility of results across pipeline runs. The nf-core/scnanoseq workflow follows best practices for analyzing single-cell and single-nuclei data, performing barcode detection and correction, genome and transcriptome read alignment, unique molecular identifier deduplication, gene and transcript quantification, and extensive quality control reporting.

**Availability and implementation:**

The source code, and detailed documentation are freely available at https://github.com/nf-core/scnanoseq and https://nf-co.re/scnanoseq under the MIT License. Documentation for the version of nf-core/scnanoseq used for this paper, including default parameters and descriptions of output files are available at https://nf-co.re/scnanoseq/1.1.0

## 1 Introduction

Advances in single-cell and single-nuclei transcriptomics have enhanced our understanding of cellular heterogeneity by enabling high-resolution gene expression analysis. Traditionally, single cell RNA sequencing (scRNA-seq) relied on short-read sequencing, which provided high base accuracy but failed to capture full-length transcripts (Byrne *et al.* 2017, Gupta *et al.* 2018, Tian *et al.* 2021, Shi *et al.* 2023). Long-read platforms from Pacific Biosciences and Oxford Nanopore Technologies (ONT) were available and capable of full-length transcript sequencing, but were limited by higher error rates and lower throughput, which complicated barcode and Unique Molecular Identifier (UMI) recovery. Recent improvements, such as ONT’s Q20+ chemistry (10X 2022c), have overcome these limitations, improving accuracy and enabling isoform-level quantification without the need for complementary short-read sequencing (10X 2022c, Prjibelski *et al.* 2023, Shi *et al.* 2023, Kumari *et al.* 2024).

Computational workflows, such as FLAMES (Tian *et al.* 2021), wf-single-cell (Oxford Nanopore Technologies 2024), and scywalker (De Rijk *et al.* 2024), support long-read-based single-cell quantification but often rely on custom tooling or *ad hoc* workflows, hindering reproducibility, limiting configurability and reporting, and complicating integration with broader bioinformatics workflows. Adapting to new data, parameters, or references can be slow, and inconsistent reporting and poor modularity can limit scalability and result comparison. To overcome these challenges, we developed nf-core/scnanoseq, a Nextflow (Di Tommaso *et al.* 2017) based pipeline within the nf-core framework (Ewels *et al.* 2020, Langer *et al.* 2025) for single-cell ONT data. It integrates open-source tools for gene- and transcript-level quantification without short-read dependency and offers genome- and transcriptome-aligned analysis. Built with Nextflow DSL 2.0 best practices, the pipeline is modular, configurable, and portable, allowing users to tailor workflows while maintaining reproducibility. These features make nf-core/scnanoseq a robust and user-friendly solution for long-read single-cell RNA sequencing.

## 2 Pipeline design and implementation

**Figure 1.**
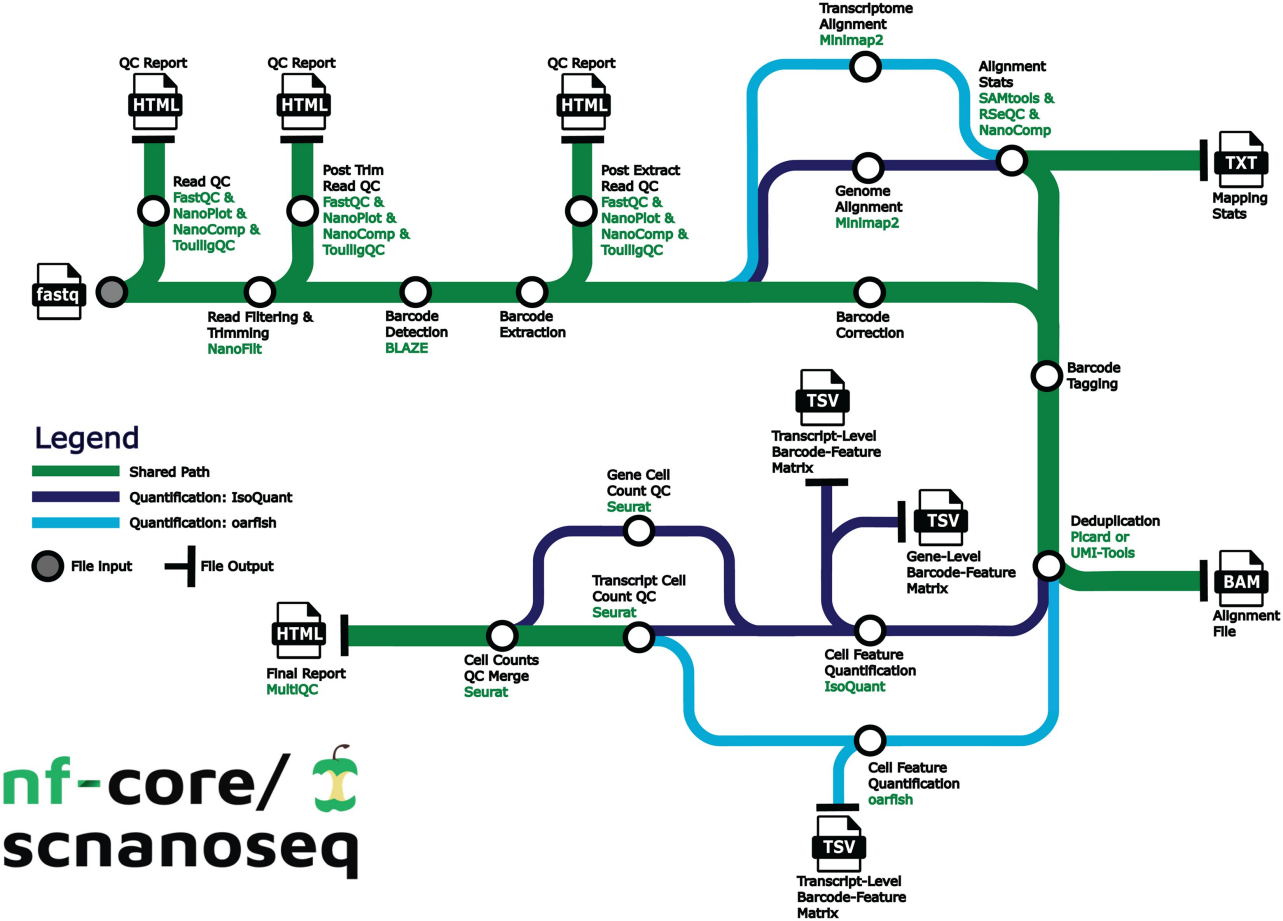
nf-core/scnanoseq workflow overview. nf-core/scnanoseq performs secondary data analysis of 10X Genomics single-cell/nuclei data derived from Oxford Nanopore sequencing. The diagram outlines the pathways to gene-level and transcript-level analysis, with file outputs noted by the file type.

nf-core/scnanoseq is built with Nextflow (Di Tommaso *et al.* 2017) DSL 2.0, ensuring modularity and simplifying future expansions. It leverages the nf-core (Ewels *et al.* 2020, Langer *et al.* 2025) framework, providing standardized guidelines and essential tools, including automated testing. The pipeline runs seamlessly on local machines, High Performance Computers (HPC), and the cloud, with built-in support for Docker and Singularity, ensuring reproducibility and portability without manual software installation. Designed as an end-to-end solution, nf-core/scnanoseq processes ONT 10X Genomics scRNA data (Fig. 1, Table 1, available as supplementary data at *Bioinformatics* online). The following sections detail its components.

### 2.1 Initial FASTQ processing

nf-core/scnanoseq accepts FASTQ files and sample metadata as input, requiring reads to contain a cellular barcode and UMI. While currently validated for use with 10X Genomics sequencing kits, its modular design allows for future support of other barcode formats. Optionally, FASTQ files can be trimmed and filtered using NanoFilt (De Coster *et al.* 2018) based on read quality and length. The unprocessed or trimmed FASTQ files are then processed with BLAZE (You *et al.* 2023) to extract uncorrected cellular barcodes and UMIs.

### 2.2 Barcode processing and alignment

Barcodes, UMIs, and additional sequences (e.g. PCR primers, TSO, poly-T tails) are extracted from reads using a custom script, leveraging raw BLAZE (You *et al.* 2023) outputs. This step generates a barcode-free FASTQ file for alignment with Minimap2 (Li 2018) and a CSV containing barcodes, UMIs, and quality scores for correction.

Barcode correction runs in parallel with alignment using a custom Python script. Barcodes are ranked by abundance and corrected if they fall within a Hamming distance of ≤2 from a whitelist barcode and have a posterior probability ≥97.5%. Corrected barcodes are updated accordingly.

After alignment and correction, a custom script tags each BAM read with raw and corrected barcodes (CR, CB), UMI (UR), and quality scores (CY, UY). The BAM file is deduplicated using UMI-Tools (Smith *et al.* 2017) or Picard MarkDuplicates (Broad Institute 2019). To optimize UMI-Tools, genome-aligned BAMs are split by chromosome, while transcriptome-aligned BAMs are split by features belonging to the same chromosome. Deduplication is optional when using IsoQuant (Prjibelski *et al.* 2023) but required for oarfish (Jousheghani *et al.* 2025).

### 2.3 Quantification

The barcode and UMI-tagged BAM files are then input for nf-core/scnanoseq quantification. The pipeline supports two methods, which can be run individually or in parallel. IsoQuant (Prjibelski *et al.* 2023) performs gene- and transcript-level quantification using genome-aligned BAMs. The “--read_groups” flag groups results by cellular barcode, generating barcode-feature matrices for downstream analysis with tertiary analysis packages, such as Seurat (Hao *et al.* 2021) or Scanpy (Wolf *et al.* 2018). Oarfish (Jousheghani *et al.* 2025) quantifies transcripts from transcriptome-aligned, UMI-deduplicated BAMs. Its outputs are further processed to produce results compatible with single-cell analysis tools, including those within this pipeline.

### 2.4 Quality control

nf-core/scnanoseq performs quality control (QC) at multiple stages: raw data, post-mapping, and post-quantification. For FASTQ files, FastQC (Andrews 2010), NanoPlot (De Coster and Rademakers 2023), NanoComp (De Coster and Rademakers 2023), and ToulligQC (Dias *et al.* 2024) provide general and long-read-specific QC across three FASTQ sets: (i) raw input, (ii) trimmed, and (iii) barcode-extracted. Reports and QC images are generated for review. For BAM files, SAMtools (Li *et al.* 2009), RSeQC (Wang *et al.* 2012), and NanoComp provide post-mapping QC. SAMtools flagstat, idxstats, and stats are run on (i) initially mapped BAMs, (ii) barcode- and UMI-tagged BAMs, and (iii) UMI-deduplicated BAMs.

Custom QC steps further extend these analyses. After quantification, Seurat (Hao *et al.* 2021) calculates single-cell metrics (e.g. cell counts, mean reads per cell, nFeature/nCount plots). A summary CSV tracks read counts across key steps. Finally, MultiQC (Ewels *et al.* 2016) compiles QC results, including custom metrics, into a single HTML report, for streamlined review.

## 3 Results

### 3.1 nf-core/scnanoseq enables reliable quantification of long-read single-cell and nuclei datasets

nf-core/scnanoseq was evaluated across three datasets: (i) 10X Genomics 3′ PBMC dataset (10X Genomics 2022c,b), (ii) 10X Genomics 5′ stage III squamous cell lung carcinoma (10X Genomics 2022a,c) (lung cancer DTCs), and (iii) You *et al.* pluripotent stem cells undergoing cortical neuronal differentiation (You *et al.* 2023) (Table 2, available as supplementary data at *Bioinformatics* online). The 3′ PMBC and 5′ lung cancer DTCs datasets included matched Illumina data for gene-level comparisons, while You *et al.* provided gene- and transcript-level quantification via BLAZE (You *et al.* 2023)-FLAMES (Tian *et al.* 2021) enabling direct comparisons at both levels.

**Figure 2.**
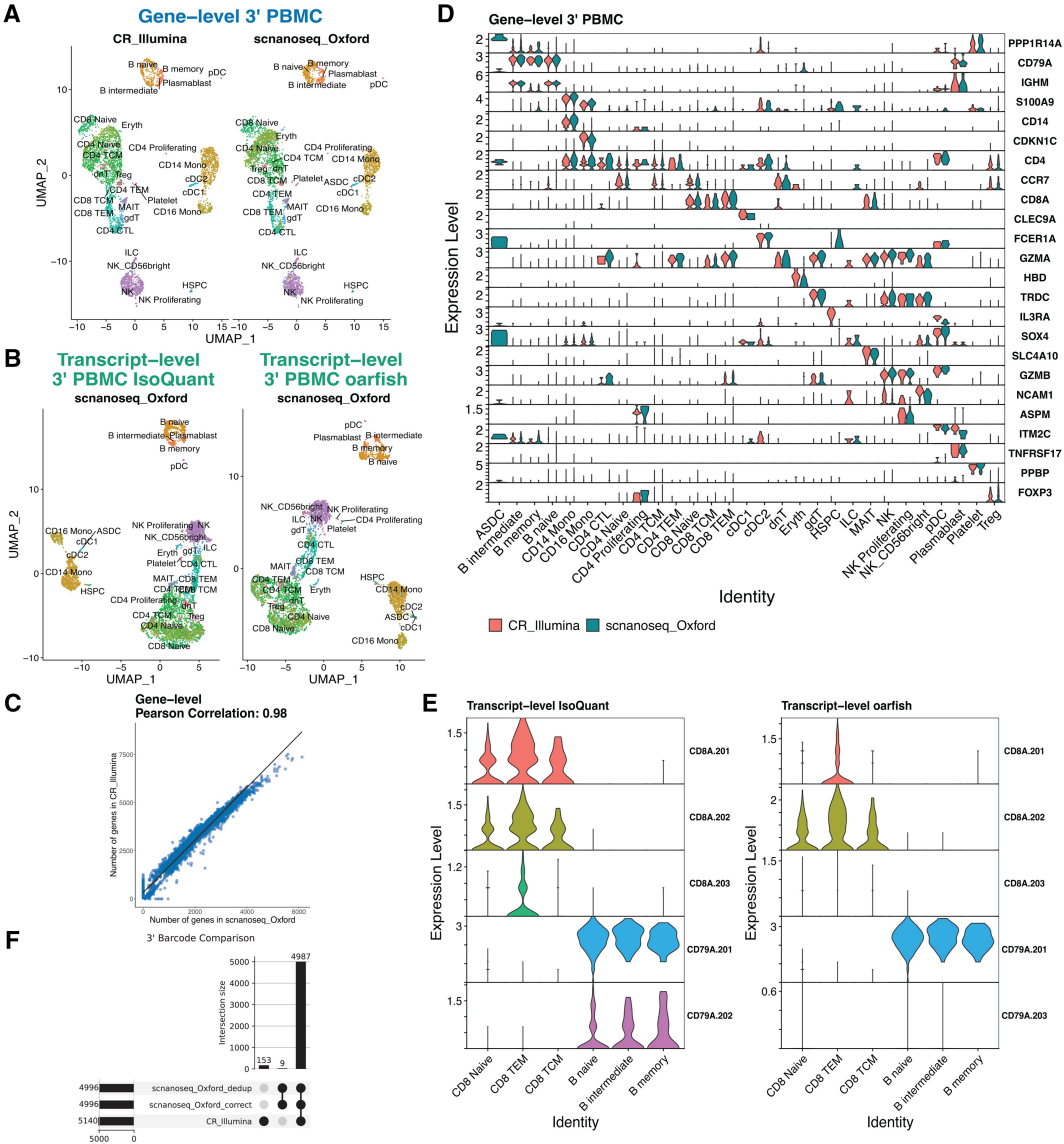
nf-core/scnanoseq validation against 3’ PBMC ground truth. UMAPs of cell clusters identified across scnanoseq and Cell Ranger [CR] short-read data at the (A) gene-level (CR and scnanoseq pipelines) and (B) trancript-level (scnanoseq pipeline only, under lsoQuant and oarfish quantifiers). (C) Gene-level scatter plot with Pearson correlation value between the number of genes detected in a cell from scnanoseq (x-axis) and CR (y-axis). (D) Gene-level expression of subset of Azimuth PBMC markers split by scnanoseq with long-read data and CR short-read data. (E) Violin plots comparing transcript-level isoform expression for *CD8A* and *CD79A* isoforms with scnanoseq using the lsoQuant and oarfish quantifiers. (F) Barcode comparison from scnanoseq at two pipelines stages (correction and post deduplication) and CR data. Bar chart at the left represents the total number of barcodes across each dataset. Bar chart at the top represents the intersection.

In the 3′ PBMC and 5′ lung cancer DTCs datasets, post-integration cell annotation with Azimuth (Hao *et al.* 2021) showed high concordance between nf-core/scnanoseq and Cell Ranger (CR) short-read data (Fig. 2A, Fig. 1A, available as supplementary data at *Bioinformatics* online), with successful cell label transfer to transcript-level data (Fig. 2B, Fig. 1B, available as supplementary data at *Bioinformatics* online). A direct comparison of detected genes per cell yielded a strong correlation (Pearson *r* = 0.98) between nf-core/scnanoseq and CR gene-level datasets (Fig. 2C, Fig. 1C, available as supplementary data at *Bioinformatics* online). Expression analysis of Azimuth markers at both gene and transcript levels revealed comparable expression profiles between nf-core/scnanoseq and CR (Fig. 2D, Fig. 1D, available as supplementary data at *Bioinformatics* online) and isoform-specific patterns across available quantifiers (Fig. 2A and B, available as supplementary data at *Bioinformatics* online). For instance, while *CD79A* is broadly expressed in B cells, *CD79A.201* is the predominant isoform (Fig. 2D and E). Similarly, *C1QB* is widely expressed in macrophages with *C1QB.201* and *C1QB.203* being the dominant isoforms over *C1QB.202* and *C1QB.204* (Fig. 1D and E, available as supplementary data at *Bioinformatics* online). These quantifications further underscore the importance of accounting for algorithm-specific behavior, as the detection—or absence—of specific isoforms can vary depending on the method chosen. For example, the isoform *CD79A.202* is detected by IsoQuant, but is absent from oarfish in the SCTransform assay due to its extremely low expression levels. Lastly, comparisons with the You *et al.* dataset (BLAZE-FLAMES) showed that nf-core/scnanoseq recovered 2–3 times more genes, transcripts and molecules per cell in the PromethION sample (ERR9958135) (Fig. 3A–D, available as supplementary data at *Bioinformatics* online). Additionally, gene, transcript and molecule count per cell remained highly correlated at both levels (Pearson *r* = 0.94–0.95).

### 3.2 Benchmark and additional validation

nf-core/scnanoseq has been extensively benchmarked and validated as shown in the Supplementary Information.

## 4 Conclusion

Here, we present nf-core/scnanoseq, a robust secondary analysis pipeline for long-read single-cell RNA-seq analysis that enables both gene- and isoform-level transcriptomic profiling through genome- and transcriptome-based quantification workflows. Fully automated, nf-core/scnanoseq streamlines preprocessing steps including alignment, deduplication, barcode correction, and tagging, while ensuring reproducibility, portability and scalability.

Unlike other long-read single-cell pipelines such as scywalker (De Rijk *et al.* 2024) and wf-single-cell (Oxford Nanopore Technologies 2024), nf-core/scnanoseq leverages the nf-core framework to deliver modular, peer-reviewed workflows supporting flexible and reproducible analysis. The use of Nextflow DSL 2.0 enables seamless expansion, allowing customization of key steps, including FASTQ trimming, filtering, and barcode whitelists. Furthermore, nf-core/scnanoseq uniquely supports two quantification methods, IsoQuant (Prjibelski *et al.* 2023) and oarfish (Jousheghani *et al.* 2025), within a unified framework, minimizing manual intervention while offering flexibility based on their specific needs.

Ongoing and planned improvements include updating supported tools like IsoQuant and BLAZE (You *et al.* 2023) as new versions are released, and optimizations to improve pipeline efficiency, particularly for IsoQuant. Emerging methods, e.g. lr-kallisto (Loving *et al.* 2025) for single-cell expression quantification, will be regularly reviewed for potential integration.

As researchers continue to adopt long-read technologies, we note that certain biological and technical considerations remain open areas of investigation. For example, gene, isoform or cell-type specific discrepancies between sequencing technologies are important considerations for study design and data interpretation. The modular design of nf-core/scnanoseq is intended to support future studies aiming to resolve such questions as sequencing technologies continue to mature. In addition, given the sensitivity of barcode correction to input read quality, we recommend using high-fidelity chemistries such as ONT’s Q20+ to minimize data loss and improve recovery of true cellular barcodes.

By combining standardized secondary analysis practices with a modular, customizable design, nf-core/scnanoseq enables accurate gene and transcript quantification while offering users the flexibility to adapt the pipeline to their specific analysis needs and preferred downstream tertiary analysis tools.

## Supplementary Material

btaf487_Supplementary_Data

## Data Availability

The source code, and detailed documentation are freely available at https://github.com/nf-core/scnanoseq and https://nf-co.re/scnanoseq under the MIT License (DOI 10.5281/zenodo.13899279).
