# Supplementary material for: scnanoseq: an nf-core pipeline for Oxford Nanopore single-cell RNA-sequencing
Source: Bioinformatics. 2025 Sep 4;41(9):btaf487. doi: 10.1093/bioinformatics/btaf487 (PMC12449243; doi:10.1093/bioinformatics/btaf487)
Supplement: btaf487_Supplementary_Data [file btaf487_supplementary_data.docx]

**scnanoseq: an nf-core pipeline for Oxford Nanopore single-cell RNA-sequencing**

**SUPPLEMENTARY MATERIALS**

**nf-core/scnanoseq tools**

| **Name** | **Tool Category** | **Required** |
| --- | --- | --- |
| Nanofilt | Filtering | No |
| BLAZE | Barcode Caller | Yes |
| Minimap2 | Alignment | Yes |
| SAMtools | Various (e.g.: Quality Control,  Reference Indexing, File Manipulation) | Yes |
| UMItools | Deduplication | No (IsoQuant),  Yes (oarfish) |
| Picard MarkDuplicates | Deduplication | No (IsoQuant),  Yes (oarfish) |
| IsoQuant | Quantification | Yes |
| oarfish | Quantification | Yes |
| Seurat | Quality Control | No |
| UCSC | File Manipulation | Yes |
| pigz | File Manipulation | Yes |
| FastQC | Quality Control | No |
| NanoComp | Quality Control | No |
| NanoPlot | Quality Control | No |
| ToulligQC | Quality Control | No |
| RSeQC | Quality Control | No |
| MultiQC | Quality Control | No |
| Custom Scripts | Barcode Extraction, Barcode Correction, Barcode Tagging,  File Manipulation | Yes |

**Supplementary Table 1:** List of tools used in nf-core/scnanoseq, categorizing their purpose in the pipeline, and noting if the tool is required within the pipeline as of v1.2.0. Any steps noted as “NO” are not required, but users are encouraged to enable any which can improve the analysis or result interpretation.

**Sample metadata**

| **sample_name** | **platform** | **tissue_type** | **source** | **read_count** | **Analysis type** |
| --- | --- | --- | --- | --- | --- |
| ERR9958135 | PromethION | cortical neuronal differentiated stem cells | You et al. | 61,967,455 | validation |
| 5' lung cancer DTCs | PromethION | lung cancer DTCs | 10X Application Note | 106,105,266 | validation and benchmarking |
| 3' PBMC | PromethION | PBMC | 10X Application Note | 129,264,682 | validation and benchmarking |
| GSM656300 | PromethION | A375 Cell Line | Shiau et al. | 105,347,205 | benchmarking |
| GSM656301 | PromethION | H2030 Cell Line | Shiau et al. | 67,436,356 | benchmarking |
| GSM656302 | PromethION | Primary RCC Tumor | Shiau et al. | 98,349,656 | benchmarking |
| GSM656303 | PromethION | RCC brain metastatis kidney tumor | Shiau et al. | 87,800,959 | benchmarking |
| GSM656304 | PromethION | Primary melanoma | Shiau et al. | 72,828,041 | benchmarking |
| GSM656305 | PromethION | Melanoma Brain metastatis | Shiau et al. | 98,363,541 | benchmarking |

**Supplementary Table 2:** Compiled sample name, platform, tissue type, data source and read count for all publicly available datasets used for benchmarking and validation of nf-core/scnanoseq.

**EXTENDED RESULTS**

**Supplemental analytical figures:**

**
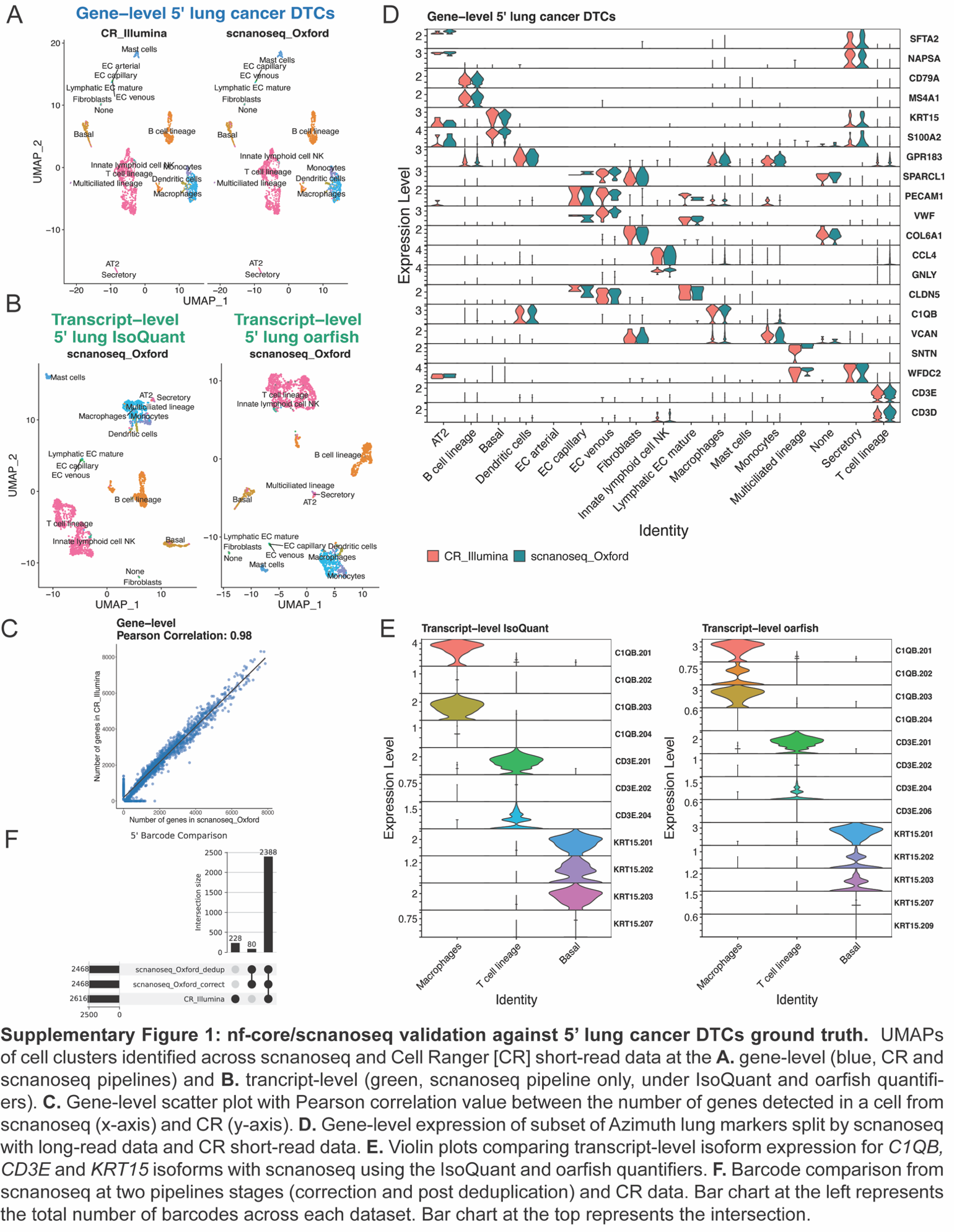
**

**
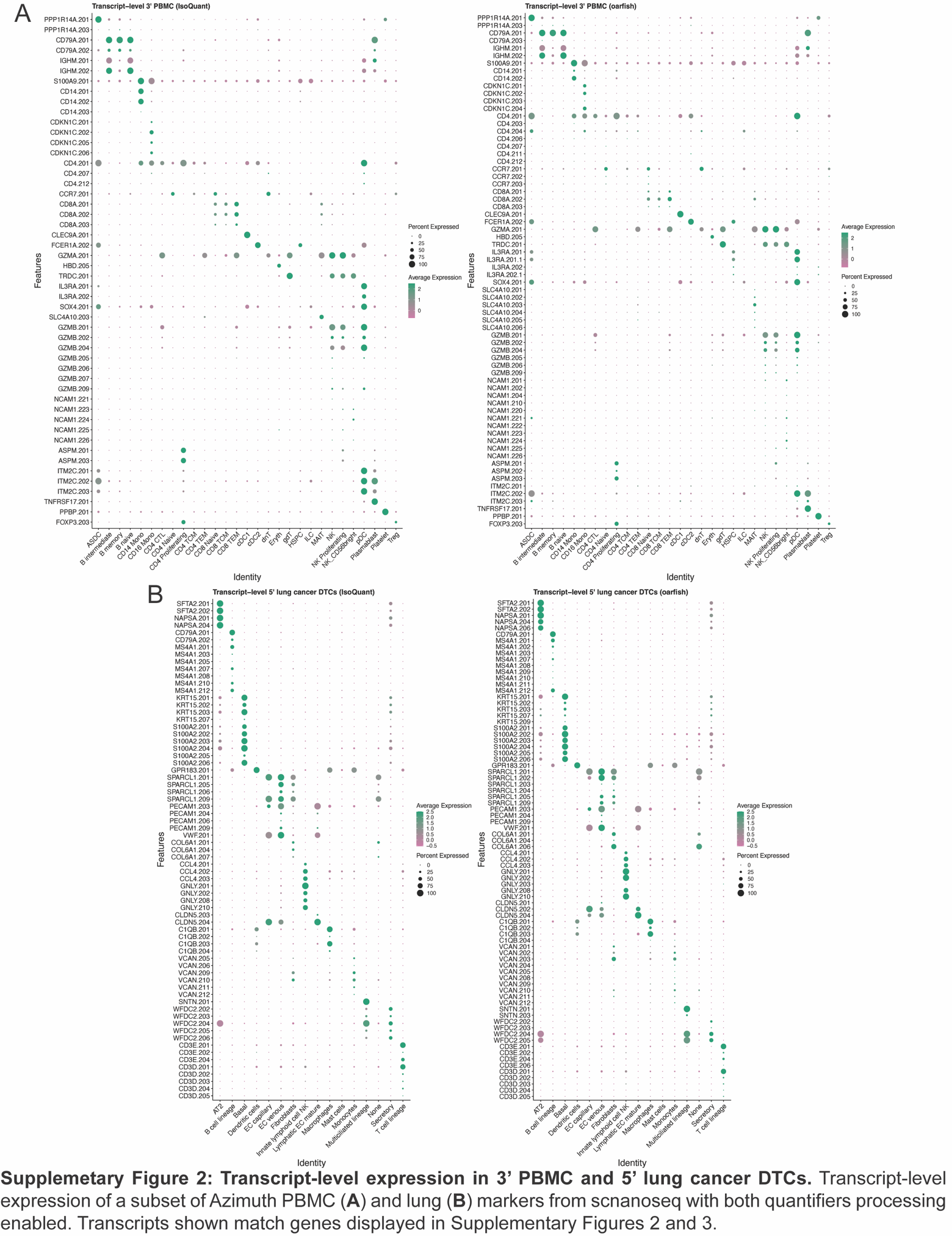

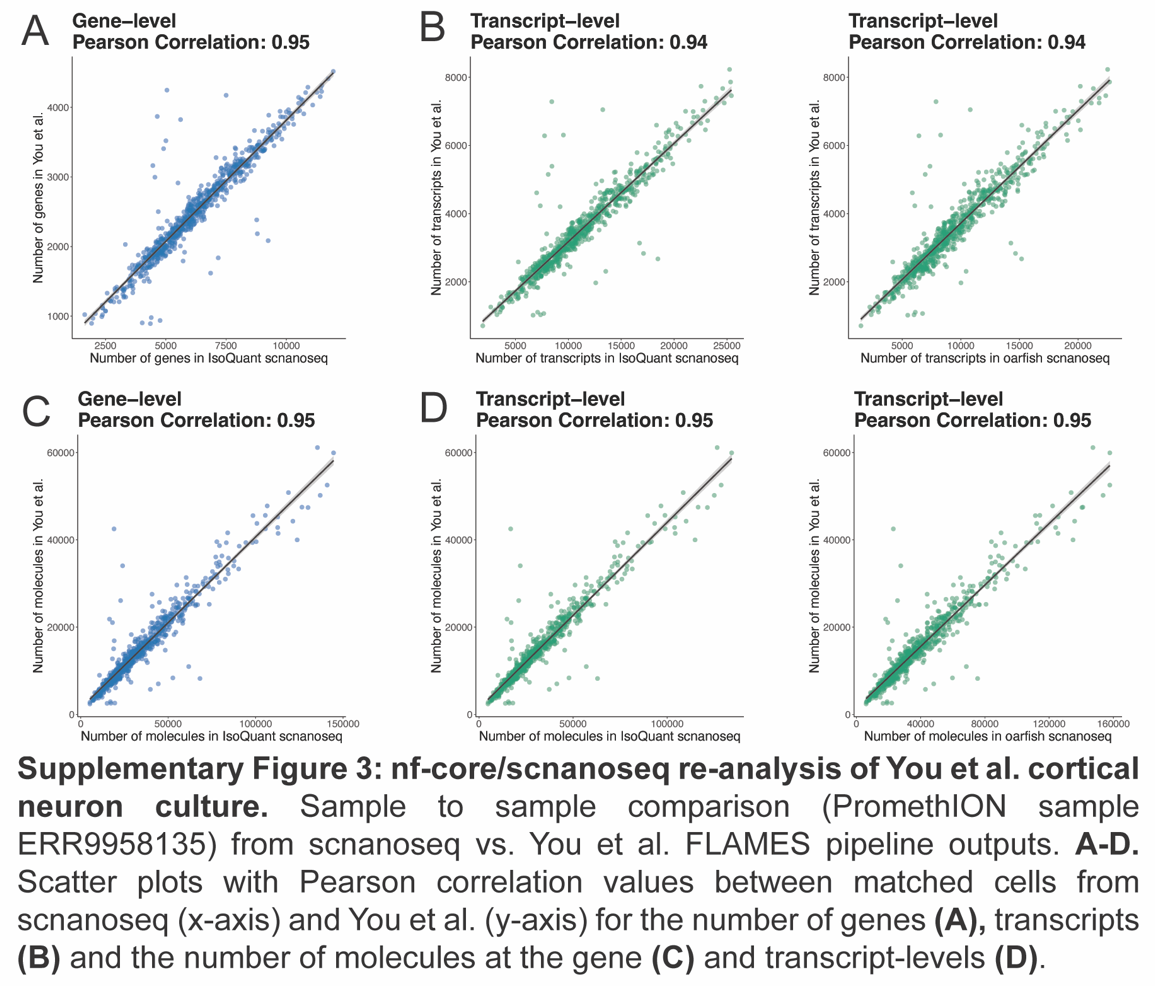

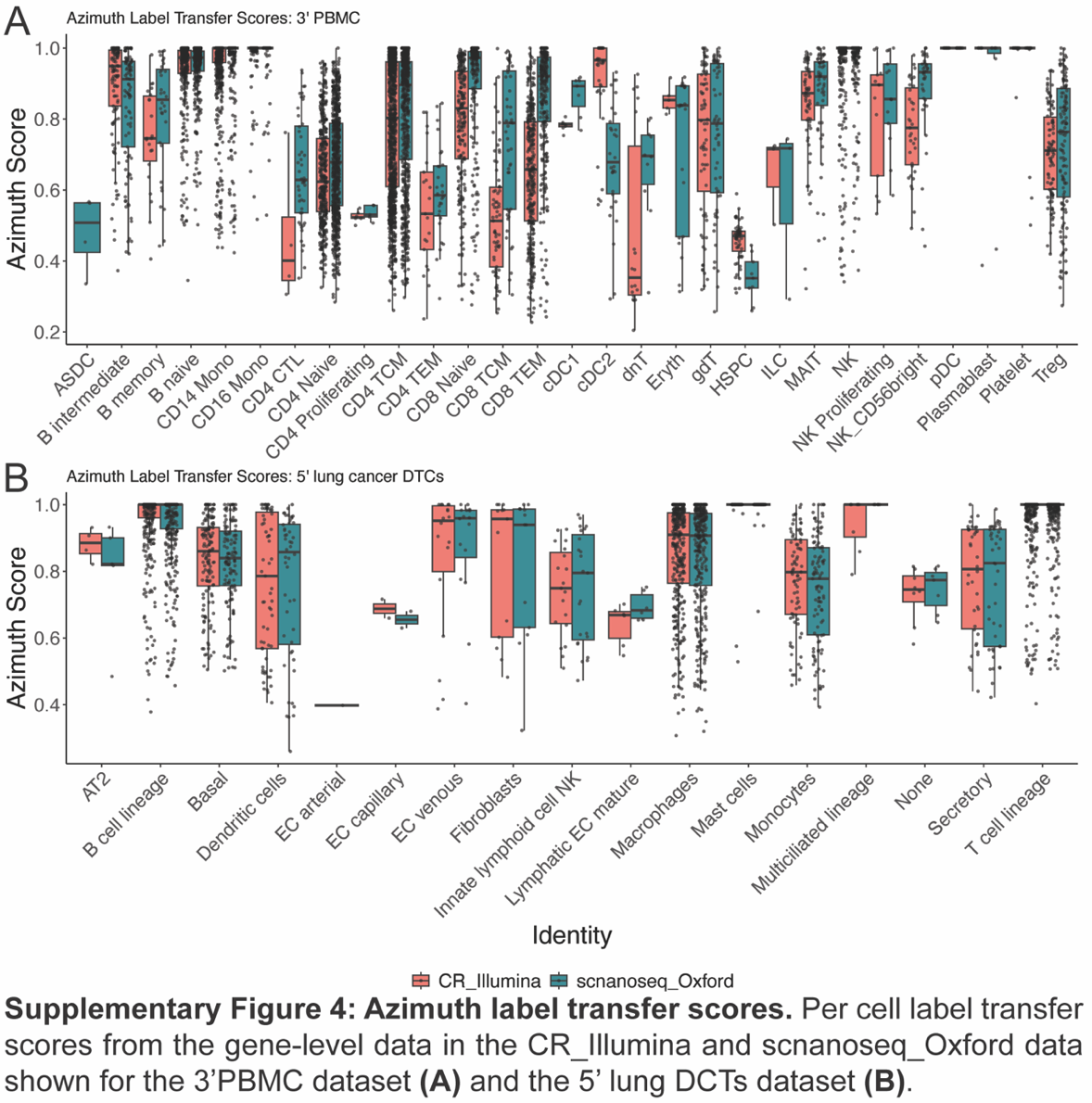
**

**Benchmark**

Ensuring an efficient end-to-end runtime is a key feature of computational pipelines. Individual pipeline steps were benchmarked using the 10X 3’ PBMC dataset to assess time and memory efficiency (**Supplementary Figure 5A**). NanoComp (De Coster and Rademakers 2023) and SAMtools (Li *et al.* 2009) were excluded from visualization. NanoComp was omitted since it aggregates results across multiple samples, leading to high memory usage in large-scale analyses without adding unique insights for single samples. While this QC step can provide additional summary statistics, its omission does not compromise essential quality control metrics, as nf-core/scnanoseq integrates key QC results into MultiQC (Ewels *et al.* 2016) alongside other QC-specific steps. SAMtools was excluded due to the number of times various subtools are called, each contributing minimally to overall runtime.

Among QC processes, pre-alignment QC steps such as FastQC (Andrews 2010), NanoPlot (De Coster and Rademakers 2023), ToulligQC (Dias *et al.* 2024) require the most time, with FastQC being the most time-consuming. To optimize execution time, all QC steps in nf-core/scnanoseq are optional, allowing users to skip individual steps or disable all QC with a single flag. This flexibility enables users to select specific QC tools instead of running all available options, such as choosing between FastQC or NanoPlot for read QC metrics reported in MultiQC. Steps marked with an asterisk (* in Supplementary Fig. 5) indicate processes that have been parallelized, with the longest subprocess runtime reported. Parallelization in nf-core/scnanoseq is implemented through file-splitting, allowing tools to process smaller chunks of data concurrently. This approach is necessary for methods that lack native multithreading support, such as NanoFilt (De Coster *et al.* 2018), as well as for multithreaded tools that still require significant runtime, such as IsoQuant (Prjibelski *et al.* 2023).

To further evaluate the impact of Nextflow (Di Tommaso *et al.* 2017) based file-splitting parallelization, analysis runtimes were assessed using the Shiau, CK., Lu, L., Kieser, R. et al. dataset (Shiau *et al.* 2023)(**Supplementary Fig. 5B**). The results indicate that most split processes complete within an hour, with the exception of IsoQuant, which has a maximum runtime of five hours. However, IsoQuant runtime is *heavily dependent on dataset depth*, and samples with greater sequencing depth than those presented in this work are expected to require longer runtimes. The results demonstrate that file-splitting parallelization effectively minimizes processing time across multiple steps in the pipeline. Additionally, users can further reduce runtime by selecting either IsoQuant or oarfish (Jousheghani and Patro 2024) for quantification rather than running both, allowing nf-core/scnanoseq to execute a single pathway instead of both quantifiers. The reported times in **Supplementary Figure** **5** represent pipeline execution runtimes and do not account for queue times on shared HPC systems, which are subject to external factors such as overall system load and compute resource availability.

**
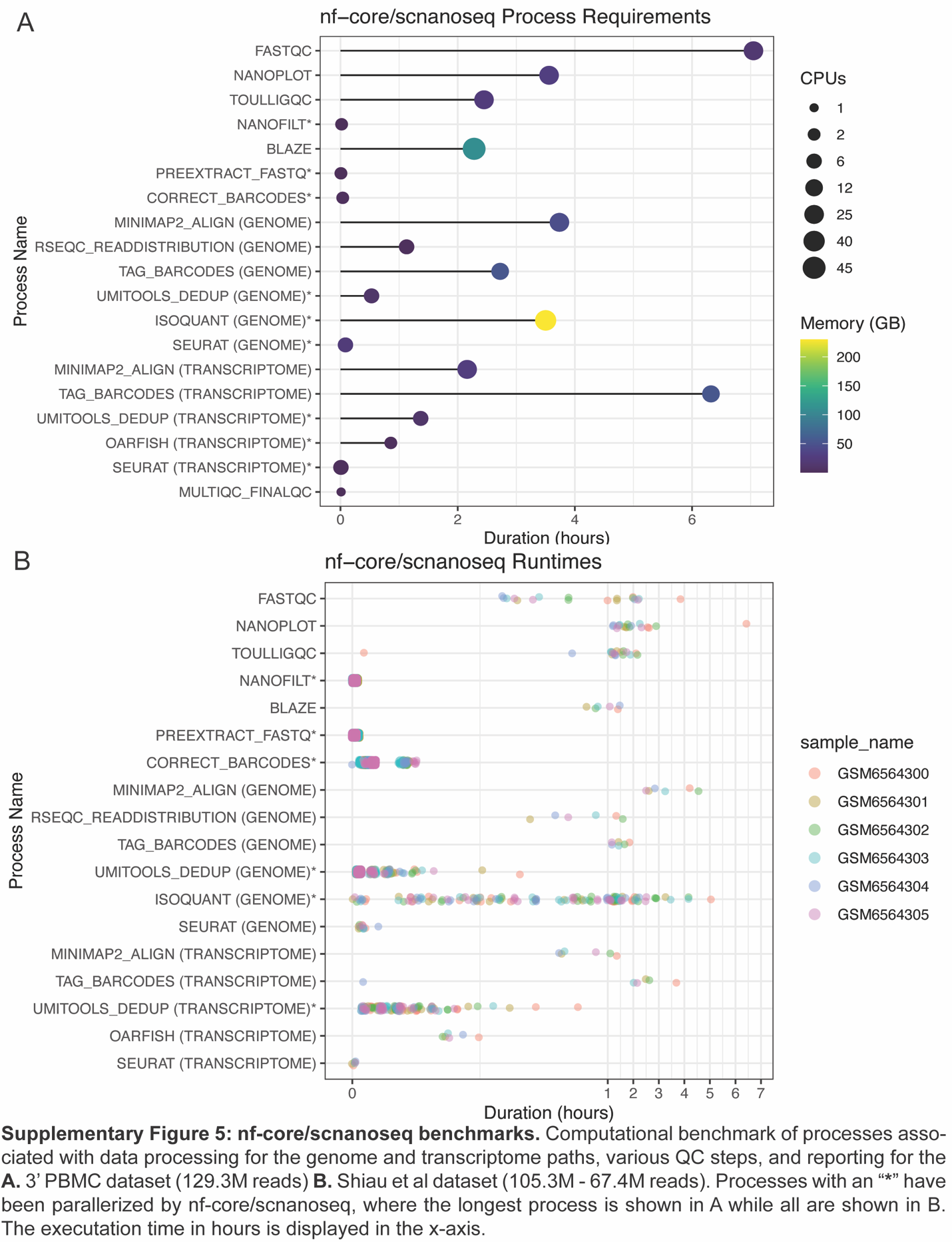
**

However, even with the file-based splitting, some processes, such as IsoQuant, can still require a large amount of memory. Much like with runtime, the memory a process requires is dependent on the read depth, with greater read depth resulting in higher memory usage. To assist users in running this pipeline, minimum system requirements for nf-core/scnanoseq are provided across a variety of read depths (**Supplementary Table 3)**. Nextflow allows for process-specific control of CPUs and memory, allowing users to configure each individual process in order to better match the resources available on their machines, *but it should be noted that running with minimum requirements will result in longer processing times*. Thus, when possible, we encourage users to increase the resources to what has been reported in Supplementary Figure 5.

| **Sequencing Kit** | **ONT Kit** | **Platform** | **Read Depth** | **Minimum Memory** | **Minimum CPUs** |
| --- | --- | --- | --- | --- | --- |
| 5’ v2 | Q20+ | PromethION | 106,105,266 | 37 GB | 5 |
| 3’ v3 | Q20+ | PromethION | 129,264,682 | 53 GB | 5 |
| 3’ v3 | LSK110 | PromethION | 61,967,455 | 36 GB | 5 |
| 3’ v3 | Q20+ | GridION | 7,521,667 | 33 GB | 5 |
| 3’ v3 | LSK110 | GridION | 3,423,062 | 37 GB | 5 |

**Supplementary Table 3:** Compiled sequencing kit, Oxford Nanopore Technologies (ONT) kit, sequencing machine information, read depth, and minimum memory and CPUs for the samples used for determining minimum system requirements for the pipeline (note: longer processing times are expected when running with minimum requirements).

**METHODS**

**Input Datasets**

To validate and benchmark nf-core/scnanoseq, we tested it on four datasets (**Supplementary Table 2):** 10X Genomics 3’ PBMC (10X 2022c, 10X 2022b) and the 5’ lung cancer DTCs (10X 2022c, 10X 2022a) as well as FASTQ files from You et al. (You *et al.* 2023) (ENA project PRJEB54718 (Leinonen *et al.* 2011)), and Shiau et al. (Edgar *et al.* 2002) (GEO accession GSE212945 (Shiau *et al.* 2023)). The latter two datasets were retrieved using nf-core/fetchngs v. 1.10.0 (Patel *et al.* 2023).

**Reference Genome, Transcriptome and Annotation**

The reference genome, transcriptome, and annotation (GTF) files used for validation matched those from the original studies: GRCh38 GENCODE release 32 (Ensembl 98) (Frankish *et al.* 2022) for the 3’ PBMC, 5’ lung cancer DTC, and Shiau et al. datasets, and GRCh38 GENCODE release 31 (Ensembl 97) for the You et al. dataset.

**nf-core/scnanoseq**

The nf-core/scnanoseq version 1.1.0 was used to analyze all datasets with default parameters, except for `min_length` (set to 500 for all runs) and `split_amount` (set to 500000 for the 3’ PBMC and 5’ lung cancer DTCs, and 1000000 for the Shiau et al. datasets). Parameters were adjusted to match the specified genome and transcriptome references, as well as the expected barcode format (3’ or 5’). For the Shiau et al. dataset, a custom `whitelist` (“737K-arc-v1.txt” from 10X Genomics) was used, in line with the authors' library preparation protocol. All runs were executed on the University of Alabama at Birmingham HPC cluster. The scripts, parameters and custom configuration files for each dataset can be found within the scnanoseq analysis repository, available at <https://github.com/U-BDS/scnanoseq_analysis/tree/main/secondary_analysis>. Detailed documentation for nf-core/scnanoseq version 1.1.0, including default parameters and description of output files, can be found at <https://nf-co.re/scnanoseq/1.1.0/>.

**Validation analysis**

All post-secondary analysis results (e.g., nf-core/scnanoseq outputs or author-reported data) were processed in a Singularity container containing downstream analytical dependencies. Analysis code is available at: <https://github.com/U-BDS/scnanoseq_analysis>. The scope of the validation analysis focused on standard downstream procedures, such as quality control, filtering, integration (where applicable), dimensionality reduction, clustering, annotation, and visualization across matched datasets. Additional analyses, such as doublet identification, were excluded to minimize downstream transformations. Below is the validation analysis for each dataset:

3’ PBMC, 5’ lung cancer DTCs: The input data included nf-core/scnanoseq outputs (gene- and transcript-level matrices from both quantifiers) and sample-matched Illumina short-read data from 10X Genomics (Cell Ranger v. 7.0.1). All analyses were conducted with Seurat (v. 4.4.0) (Hao *et al.* 2021) in R (v. 4.3.2). Quality control removed low-quality cells based on criteria: nFeature > 500 (gene), nFeature > 800 (transcript), nCount > 200 (gene, transcript), percent mitochondrial < 10 (gene, transcript). Data was normalized with SCTransform V2 (`vars.to.regress` set to percent mitochondrial), and integration was performed with Harmony (v. 1.2.0) (Korsunsky *et al.* 2019) for gene-level data (nf-core/scnanoseq and CR_Illumina). Transcript-level data from nf-core/scnanoseq was processed separately. Additionally, Azimuth (v. 0.4.6) PBMC (Stuart *et al.* 2019) and lung (Sikkema *et al.* 2023) reference sets were implemented to annotate the cell types and the cell annotations from the gene-level dataset were transferred to the transcript-level data based on matching barcodes. Dimensionality reduction and clustering were executed with the following parameters: dims = 25, resolution = 0.7 (gene, PBMC), dims = 27, resolution = 0.6 (gene, lung), dims = 25, resolution = 0.8 (transcript, PBMC), dims = 25, resolution = 0.6 (transcript, lung). The Azimuth cell annotation was set to the predicted “celltype.I2” (PBMC) and “ann_level_3” (lung), and their per-cell scores are shown in **Supplementary Figure 4**. The gene-level scatter plots illustrating the number of genes detected were generated between all cells detected in the input data (pre-quality filtering), and the correlation values were computed with the Pearson correlation coefficient. A representative subset of Azimuth PBMC and lung markers were selected for visualization across nf-core/scnanoseq (gene, transcript) and CR_Illumina (gene).

Transcript-level plots for IsoQuant and oarfish isoforms were generated on all retained isoforms in the SCTransform assay following data processing described above.

UpSet plots were generated with the upsetplot package (v. 0.9.0) in Python (v. 3.12.0), using unique barcodes from the barcode-corrected and UMI-deduplicated BAM files. SAMtools (v.1.12) was used to extract tagged barcodes from the BAM outputs of nf-core/scnanoseq.

You et al.: The input data contained the outputs of nf-core/scnanoseq (gene and transcript-level matrices) and the author provided matrices (gene and transcript-level matrices). Each pipeline dataset was separately processed with quality and control filtering low-quality cells based on nFeature > 100 (gene, nf-core/scnanoseq and You et al.), nFeature > 700 (transcript, nf-core/scnanoseq and You et al.), percent mitochondrial < 20 (gene, transcript, nf-core/scnanoseq only). No percent mitochondrial filtering was performed in the You et al. data provided by the authors due to the lack of matches between known mitochondrial gene and transcript IDs in this dataset. The data was normalized with SCTransform V2 and dimensionality reduction and clustering were executed with the following parameters: dims = 6, resolution = 0.6 (gene, transcript, nf-core/scnanoseq), dims = 6, resolution = 0.7 (gene, transcript, You et al.). Data visualization and reporting were focused on the PromethION sample (ERR9958135), with the scatter plots illustrating the number of genes or transcripts and the number of molecules. In addition, each scatter plot displays the Pearson correlation coefficient calculations.

**AVAILABILITY OF DATA AND MATERIALS**

nf-core/scnanoseq is available at <https://github.com/nf-core/scnanoseq> under the MIT License. Documentation is available at <https://nf-co.re/scnanoseq>. The downstream analytical code for validation analysis is available at <https://github.com/U-BDS/scnanoseq_analysis>. All dataset sources have been disclosed under the methods section for each respective dataset.

**REFERENCES**

10x Genomics*. 3k Human Squamous Cell Lung Carcinoma DTCs, Chromium X*. Universal 5' Gene Expression dataset analyzed using Cell Ranger 7.0.1, 10x Genomics, (2022, October 22).

10x Genomics*. 5k Human PBMCs, 3' v3.1, Chromium Controller*. Universal 3' Gene Expression dataset analyzed using Cell Ranger 7.0.1, 10x Genomics, (2022, October 22).

10x Genomics*. Application Note - alternative transcript isoform detection with single cell and spatial resolution*. Document Number LIT000194, 10x Genomics, (2022, October 22).

Andrews S. FastQC: A quality control tool for high throughput sequence data [online]. 2010.

De Coster W, D'Hert S, Schultz DT *et al.* NanoPack: Visualizing and processing long-read sequencing data. *Bioinformatics* 2018;**34**:2666-9.

De Coster W, Rademakers R. NanoPack2: Population-scale evaluation of long-read sequencing data. *Bioinformatics* 2023;**39**.

Di Tommaso P, Chatzou M, Floden EW *et al.* Nextflow enables reproducible computational workflows. *Nat Biotechnol* 2017;**35**:316-9.

Dias K, Laffay B, Ferrato-Berberian L *et al.* toulligQC. GenomiqueENS. 2024.

Edgar R, Domrachev M, Lash AE. Gene Expression Omnibus: NCBI gene expression and hybridization array data repository. *Nucleic Acids Res* 2002;**30**:207-10.

Ewels P, Magnusson M, Lundin S *et al.* MultiQC: Summarize analysis results for multiple tools and samples in a single report. *Bioinformatics* 2016;**32**:3047-8.

Frankish A, Carbonell-Sala S, Diekhans M *et al.* GENCODE: Reference annotation for the human and mouse genomes in 2023. *Nucleic Acids Research* 2022;**51**:D942-D9.

Hao Y, Hao S, Andersen-Nissen E *et al.* Integrated analysis of multimodal single-cell data. *Cell* 2021;**184**:3573-87 e29.

Jousheghani ZZ, Patro R. Oarfish: Enhanced probabilistic modeling leads to improved accuracy in long read transcriptome quantification. *bioRxiv* 2024.

Korsunsky I, Millard N, Fan J *et al.* Fast, sensitive and accurate integration of single-cell data with Harmony. *Nat Methods* 2019;**16**:1289-96.

Leinonen R, Akhtar R, Birney E *et al.* The European Nucleotide Archive. *Nucleic Acids Res* 2011;**39**:D28-31.

Li H, Handsaker B, Wysoker A *et al.* The Sequence Alignment/Map format and SAMtools. *Bioinformatics* 2009;**25**:2078-9.

Patel H, Beber ME, Joshi E *et al.* nf-core/fetchngs: nf-core/fetchngs v1.10.0 - manganese monkey (1.10.0). 2023.

Prjibelski AD, Mikheenko A, Joglekar A *et al.* Accurate isoform discovery with IsoQuant using long reads. *Nat Biotechnol* 2023;**41**:915-8.

Shiau CK, Lu L, Kieser R *et al.* High throughput single cell long-read sequencing analyses of same-cell genotypes and phenotypes in human tumors. *Nat Commun* 2023;**14**:4124.

Sikkema L, Ramírez-Suástegui C, Strobl DC *et al.* An integrated cell atlas of the lung in health and disease. *Nature Medicine* 2023;**29**:1563-77.

Stuart T, Butler A, Hoffman P *et al.* Comprehensive integration of single-cell data. *Cell* 2019;**177**:1888-902 e21.

You Y, Prawer YDJ, De Paoli-Iseppi R *et al.* Identification of cell barcodes from long-read single-cell RNA-seq with BLAZE. *Genome Biol* 2023;**24**:66.
